# NCX-DB: a unified resource for integrative analysis of the sodium calcium exchanger super-family

**DOI:** 10.1186/s12868-018-0423-2

**Published:** 2018-04-13

**Authors:** Katrin Bode, Damien M. O’Halloran

**Affiliations:** 1 Department of Biological Sciences, The George Washington University, Science and Engineering Hall 6000, 800 22nd St. N.W., Washington, DC 20052 USA; 2 Institute for Neuroscience, The George Washington University, 636A Ross Hall, 2300 I St. N.W., Washington, DC 20052 USA

**Keywords:** NCX, NCKX, NCLX, Sodium calcium exchanger, Database, Antiporter

## Abstract

Na^+^/Ca^2+^ exchangers are low-affinity high-capacity transporters that mediate Ca^2+^ extrusion by coupling Ca^2+^ efflux to the influx of Na^+^ ions. The Na^+^/Ca^2+^ exchangers form a super-family comprised of three branches each differing in ion-substrate selectivity: Na^+^/Ca^2+^ exchangers (NCX), Na^+^/Ca^2+^/K^+^ exchangers, and Ca^2+^/cation exchangers. Their primary function is to maintain Ca^2+^ homeostasis and play a particularly important role in excitable cells that experience transient Ca^2+^ fluxes. Research into the role and activity of Na^+^/Ca^2+^ exchangers has focused extensively on the cardio-vascular system, however, growing evidence suggests that Na^+^/Ca^2+^ exchangers play a key role in neuronal processes such as memory formation, learning, oligodendrocyte differentiation, neuroprotection during brain ischemia and axon guidance. They have also been implicated in pathologies such as Alzheimer’s disease, Parkinson’s disease, Multiple Sclerosis and Epilepsy, however, a clear understanding of their mechanism during disease is lacking. To date, there has never been a central resource or database for Na^+^/Ca^2+^ exchangers. With clear disease relevance and ever-increasing research on Na^+^/Ca^2+^ exchangers from both model and non-model species, a database that unifies the data on Na^+^/Ca^2+^ exchangers is needed for future research. NCX-DB is a publicly available database with a web interface that enables users to explore various Na^+^/Ca^2+^ exchangers, perform cross-species sequence comparison, identify new exchangers, and stay-up to date with recent literature. NCX-DB is available on the web via an interactive user interface with an intuitive design, which is applicable for the identification and comparison of Na^+^/Ca^2+^ exchanger proteins across diverse species.

**Electronic supplementary material:**

The online version of this article (10.1186/s12868-018-0423-2) contains supplementary material, which is available to authorized users.

## Background

The sodium calcium exchanger superfamily of antiporters function to maintain calcium homeostasis. This superfamily is composed of the Na^+^/Ca^2+^ exchangers (NCX) which couple the extrusion of 1 Ca^2+^ ion with the influx of 3 Na^+^ ions, the Na^+^/Ca^2+^/K^+^ exchangers (NCKX) which exchange 1 Ca^2+^ ion and 1K^+^ ion in exchange for 4 Na^+^ ions, and Ca^2+/^Cation exchangers (also called NCLX) which couple the extrusion of 1 Ca^2+^ ion with the influx of either 3 Na^+^ or 3 Li^+^ ions [1–11]. The Na^+^/Ca^2+^ exchanger super-family is highly conserved across species in both invertebrate and vertebrate organisms [12–16]. The activity of the exchanger and rate of calcium extrusion is largely dependent on intracellular calcium levels. Studies of NCX in retinal cells of *Drosophila melanogaster* revealed that forward mode activity is initiated when cytosolic calcium levels reach 500 nM [17]. During high intracellular Na^+^ concentrations, Na^+^/Ca^2+^ exchangers will reverse by initiating calcium influx [9, 18]. For instance, reverse-mode NCX plays a role in regulating glutamatergic glial transmission in rat cortical astrocytes [19]: during resting state in cortical astrocytes, the Na^+^/K^+^ ATPase pump regulates cellular Na^+^ homeostasis by extruding Na^+^ and the Plasma Membrane Ca^2+^ ATPase pump regulates cellular calcium homeostasis by extruding calcium [19]; however, upon mechanical stimulation, an increase in intracellular Na^+^ causes NCX to function in reverse mode, which mediates calcium entry into astrocytes, and increase in intracellular calcium stimulates glutamatergic gliotransmission between astrocytes and neurons [19]. Similarly in Purkinje neurons, an increase in intracellular sodium triggers NCX to function in the reverse mode [20]. Reverse mode NCX also plays a role in astrogliosis whereby astrocytes proliferate and migrate into areas of neuronal damage [21]. Consequently, attenuation of NCX activity via a pharmacological inhibitor results in decreased calcium transients within astrocytes following mechanical injury and stimulation [21]. Although they can facilitate bidirectional transport, under normal physiological conditions, Na^+^/Ca^2+^ exchangers function primarily in the forward direction by coupling the extrusion of calcium with the influx of sodium ions [22]. Under pathophysiological conditions is has been shown that NCX can play a neuroprotective role. Ischemic preconditioning is a neuroprotective mechanism in which a brief ischemia protects the brain from a subsequent lethal insult. During a non-injurious episode of brain ischemia it was shown that both NCX1 and NCX3 were up-regulated where they conferred neuroprotective roles, and knockdown of NCX1 and NCX3 reversed this neuroprotective effect [23, 24]. Furthermore, increased expression of NCX3 was observed after a prolonged harmful ischemic episode (ischemic post-conditioning), where again it was shown to confer a neuroprotective effect [23]. More recently it has been shown that sumoylation of the lysine at position 590 of NCX3 may be a key determinant for enhancing ischemic preconditioning-induced neuroprotection [25]. Thus, during both normal and pathophysiological conditions, NCX plays a central role in homeostasis by regulating Ca^2+^ exchange.

Genetic studies of NCX in the nervous system, have revealed a role for NCX3 in the expression of myelin marker genes: CNPase and myelin basic protein [26]. Moreover, *ncx3*^−*/*−^ mice exhibit reduced spinal cord size, thereby implicating NCX3 in spinal cord formation [26]. NCX3 is also expressed in the CA1 region of the hippocampus [27]. In *ncx3*^−*/*−^ knockout mice, basal intracellular calcium levels are elevated and presynaptic intracellular calcium levels exhibit a slower decline after depolarization [27]. The NCX type exchanger, NCX2, is also expressed in CA1 pyramidal neurons [28]. In *ncx2*^−*/*−^ knockout mice, clearance of intracellular calcium is significantly delayed at the presynaptic site of CA1 neurons [28] and consequently, *ncx2*^−*/*−^ knockout mice show significantly enhanced performance in learning and memory behaviors [28]. NCX1 has been shown to regulate neurite outgrowth in cortical neurons via modulation of ER calcium content and PI3K/Akt signaling [29], and more recently research in *Caenorhabditis elegans* has revealed a role for the NCLX-type exchanger NCX-9 in regulating asymmetric patterning of a motor circuit by engaging Netrin and heparan sulfate signaling effectors [30].

Na^+^/Ca^2+^ exchangers are widely expressed across the nervous system (as well as many other tissues and cell types) where they function in development, learning, memory formation, and motor function. They have also been implicated in several disease states of the nervous system such as Alzheimer’s disease, Parkinson’s disease, Multiple Sclerosis and Epilepsy, however, the exact role and mechanism of Na^+^/Ca^2+^ exchangers during disease is still unclear [31–40]. The field of researchers that study Na^+^/Ca^2+^ exchangers encompass many different branches of biology and employ diverse model systems to address various aspects of their biology [41]. In order to facilitate the exchange of data and further the field of Na^+^/Ca^2+^ exchange biology, there is a clear need for a central database that unifies Na^+^/Ca^2+^ exchanger data across species to facilitate analysis and discovery. NCX-DB is the first database of Na^+^/Ca^2+^ exchangers, and represents a central resource for discovery, cross-species comparison, while also providing a portal to recent literature in the field of Na^+^/Ca^2+^ exchangers.

## Construction and content

**Fig. 1:**
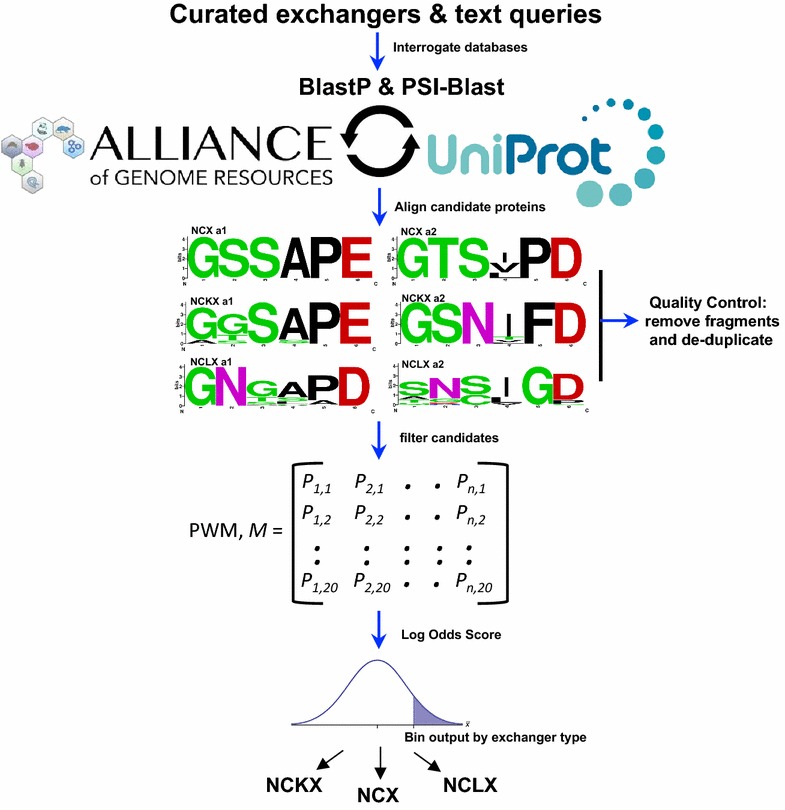
NCX-DB data workflow. Candidate exchangers were sourced from UniProt as well as the animal model organism databases that comprise the Alliance of Genome Resources. These model organism databases are: WormBase, XenBase, FlyBase, the Zebrafish Information Network, Mouse Genomics Informatics, and the Rat Genome Database. The resulting candidate exchangers were then aligned and filtered through a Position Weighted Matrix (PWM) to return a log odds score used to classify exchangers by subtype

Data contained within NCX-DB was mined from UniProt (ver. June 2017) [42] as well as the animal model organism databases that comprise the Alliance of Genome Resources (http://www.alliancegenome.org/). These are: WormBase (ver. WS260) [43], XenBase (ver. 4.5.0) [44], FlyBase (FB2017_04) [45], the Zebrafish Information Network (ZFIN: https://zfin.org/), Mouse Genomics Informatics (http://www.informatics.jax.org/), and the Rat Genome Database (https://rgd.mcw.edu/). By including the Alliance Genomes databases we could include all the major model organisms, and we chose UniProt as it is considered the most comprehensive protein database because it includes literature based curation in its functional annotations. Candidate exchangers for NCX-DB BLAST database were mined using text-based queries that relied upon literature based annotations and also by using BlastP and psi-Blast searches of downloaded genomes from each database (Fig. 1 and Table 1). Candidate exchangers were then aligned using MUSCLE [46] and filtered through a Position Weighted Matrix (PWM) based upon known sodium calcium exchanger transport repeats (α1 and α2) [6, 47–52]. To design the PWM, a Position Probability Matrix (PPM) was determined from the residue frequency of each site of aligned known sodium calcium exchangers. The PPM (*M*) was then used to calculate a PWM, *M’*, which was generated by transforming the matrix with a background model, *b*_*i*_, that assumes equal residue frequency, as follows:

$$M' = \log 2(Mij/bi)$$

where, *M*_*ij*_ is the probability of residue *i* at position *j* in *M above*.

**Table 1:** Summary of NCX-DB BLAST database content

Classification	Number of entries
Sodium Calcium Exchangers	483
Total amino-acids (a.a)	348,524
Average protein length (a.a)	723
Largest protein length (a.a)	1283
Smallest protein length (a.a)	242
Acidic residues	56,677
Basic residues	35,288
Neutral residues	56,677
Most common amino acid	Leucine (33,547)
Least common amino acid	Tryptophan (6031)

Using this matrix, candidate exchangers were filtered by exchanger type based upon their Log Odds score. The alpha-repeats have been shown to form a diamond shaped transport vestibule which facilitates ion transport [53]. The filtered data was then converted to protein databases using BLAST [54] local executables (ftp://ftp.ncbi.nlm.nih.gov/blast/executables/blast+/LATEST/) and organized in various databases by taxonomy and exchanger subtype. BLAST searches are ran on the server-side using PHP scripts and served asynchronously using Ajax (http://api.jquery.com/jquery.ajax/) and jQuery (http://jquery.com/). FASTA formatted sequences can be retrieved at the NCX-DB BLAST page by using the NCBI Entrez Programming Utilities (https://www.ncbi.nlm.nih.gov/books/NBK25501/). Accession numbers can be entered as a comma separated list and retrieved remotely. The following parameters are implemented for BlastP searches using NCX-DB: initial *word_size* match is 3; the minimum threshold score to add a word to the BLAST lookup table is 11; the composition-based score adjustment is defined as per [55]; the heuristic value (in bits) for the final gapped alignment is 25; and the window size for multiple hits is set to 40.

The un-filtered exchanger data is also available at NCX-DB under the *Browse* link. The *Browse* data includes fragments, and is comprised of predicted exchangers collected into NoSQL tables and converted into searchable JavaScript object notation (JSON) objects. Data is visualized using jQuery table plugins. The user can select the number of entries to view per page (default is 25), and search the data by species (common or Latin name), or exchanger. The data can also be ordered into groups by clicking on the various table headers. Entries within the browsing tables also return URLs for each entry which link to various databases that were used to source the exchangers within NCX-DB.

In order to facilitate the discovery of novel exchangers or characterize predicted exchangers, NCX-DB contains a tool under the *Predict* link which searches a user supplied protein sequence for candidate sodium calcium exchanger transport repeats. To search for candidate exchanger motifs, NCX-DB will scan a user supplied sequence for a fuzzy match to the motif; if a match is found, NCX-DB then uses the PWM described above, to sum the corresponding log likelihood values at each residue to return a score for the candidate exchanger motif. The *Predict* tool provides a graphical sequence output highlighting the position of the alpha repeats within the candidate exchanger, and also a chart depicting residue frequency. The sequence graphical output was modified from the neXtProt protein sequence viewer available at biojs.io, and the chart is generated using a JavaScript function to convert the residue frequency into a JSON object for canvas.js (https://canvasjs.com/).

To examine the phylogenetic relationship between exchanger subtypes, the user can visit the *Phylogeny* page and view radial unrooted phylogenetic trees for each exchanger subtype (i.e. NCX, NCKX, and NCLX). Protein sequences for each tree was aligned using MUSCLE [46, 56] and the resulting alignments were used to generate Newick formatted trees using PhyML [57, 58]. Newick trees were converted to phyloXML (http://www.phyloxml.org/) and visualized using jsPhyloSVG [59]. XML files for each tree was edited to include clade and phylum specific features so as to make the experience more interactive. Each tip on the tree is also linked to other databases where the user can obtain more information and background on each exchanger.

**Fig. 2:**
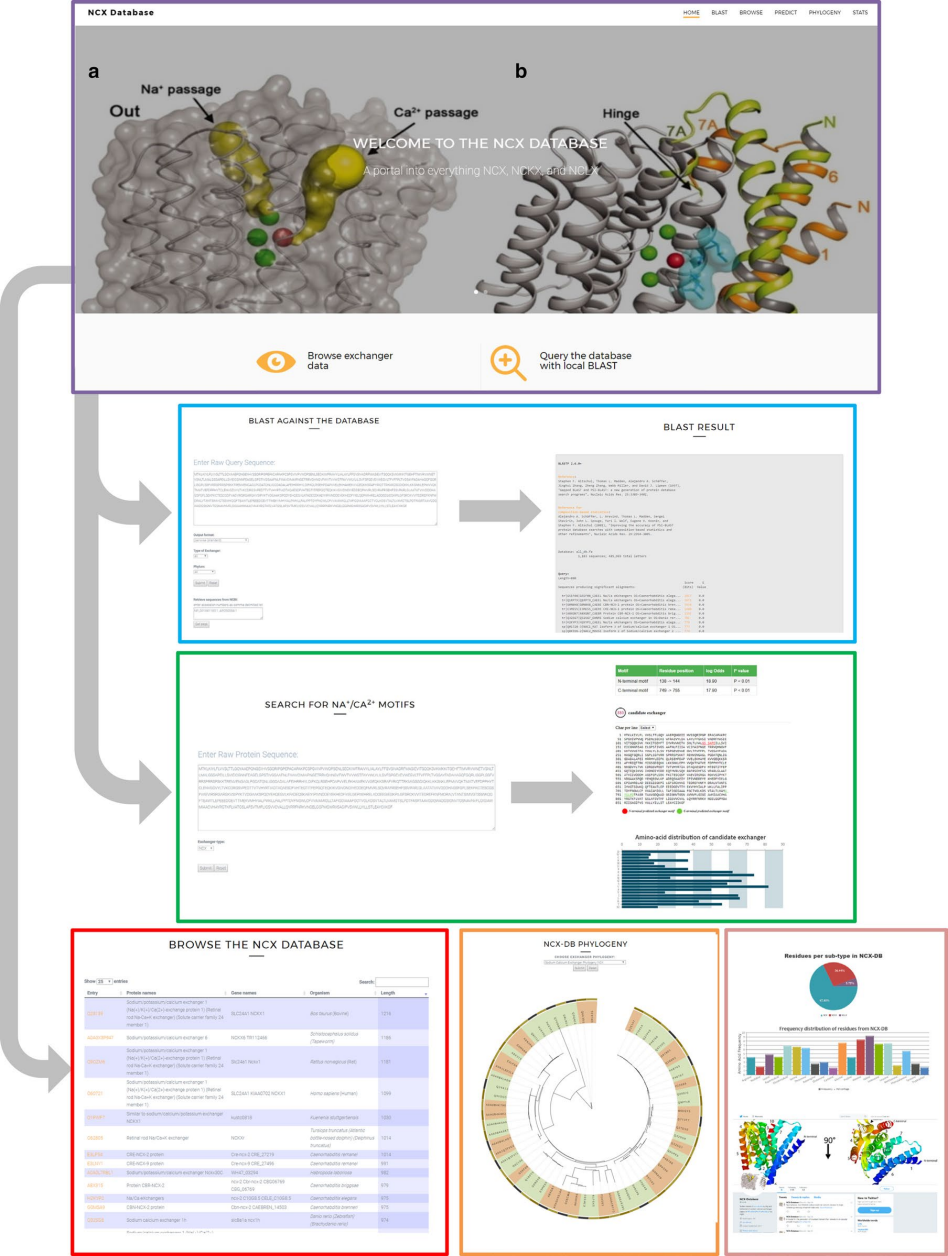
NCX-DB interface and features. The landing page of NCX-DB and various features are shown. From the landing page, users can navigate through various links to browse NCX-DB in table form, query NCX-DB via local BLAST executables, examine phylogenetic relationships, and characterize novel exchangers using the *Predict* tool. Users can view recent literature and developments in the field of Na^+^/Ca^2+^ exchange biology, and also link to other databases through NCX-DB. NCX-DB is designed with an interactive graphical user interface which is intuitive and provides seamless exploration and discovery options

Finally, NCX-DB also contains a *Stats* page which details the number of exchangers, exchanger sub-types, and residue counts contained within the database, as well as a Twitter feed for NCX-DB (Twitter handle: @ncxdb). The Twitter account for NCX-DB serves as an information service about all things related to sodium calcium exchangers (meetings, societies, etc.) while also providing data related to NCX-DB. In addition the Twitter account has been configured to serve as a twitterbot that captures the RSS feed on searches related to sodium calcium exchangers from PubMed (https://www.ncbi.nlm.nih.gov/pubmed) as well as the pre-print servers: bioRxiv (https://www.biorxiv.org/) and arXiv (https://arxiv.org/). Therefore, visitors can keep up-to-date with the literature related to sodium calcium exchangers by following @ncxdb or by visiting NCX-DB. The front-end for NCX-DB was written in JavaScript and uses Bootstrap (http://getbootstrap.com/) and jQuery (http://jquery.com/) to provide an intuitive graphical user interface (Fig. 2).

## Utility and discussion

NCX-DB represents the first centralized database for sodium calcium exchangers. Data contained within NCX-DB is mined from highly annotated databases so as to collate the most up to date and informative information for each exchanger across the main model organisms. On the landing page, the user can explore NCX-DB using the links along the top panel or by clicking on the graphical icons under the banner (Fig. 2). The same navigation menu along the top panel, can be viewed on all NCX-DB pages. The first link on the menu brings the user to the BLAST page for NCX-DB, where the user can input raw protein sequence data and perform BLAST searches of a query sequence against NCX-DB. Various drop-down menus permit taxonomic as well as exchanger specific BLAST searches. The user can also visualize the results in a variety of different formats, although the pairwise representation is set as the default. Sequences can also be remotely retrieved from The National Center for Biotechnology Information (NCBI) by simply entering an accession number in the textbox under *Retrieve sequences from NCBI*. Any number of accession numbers can be entered, however, for two or more, the accession numbers must be separated by a comma. To browse raw exchanger data, the user can click on the *Browse* link to view a table of predicted exchanger entries. The user can change the number of entries viewed per page in the table from 25 (default) to 100. By clicking on the table headers, the user can sort the data by heading for example, species, gene name, or exchanger length. Search terms can also be entered into the Search box at the top of the table to dynamically sort entries that contain search terms. As well as providing an overview of each entry, the Browsing tables also link to various databases which were used to source the exchangers within NCX-DB, therefore visitors can explore and learn more about specific exchangers by clicking on links in the first column of the browse table.

NCX-DB also provides facilities to characterize and search for new exchangers. To characterize candidate exchangers, users can use the *Predict* tool under the Predict link in the header menu. Users can enter the raw protein sequence of a candidate exchanger and the *Predict* tool at NCX-DB will scan the user supplied sequence for matches to exchanger specific domains. The *Predict* tool at NCX-DB returns a tabulated output with the position (in residues) of the candidate motifs and their resulting log odds score that are determined by using the PWM described above. In addition, a graphical output of the user supplied input is returned that illustrates the N terminal and C terminal candidate exchanger motifs highlighted in red and green respectively, as well as a chart of the amino-acid frequency distribution for the user-inputted sequence. Using this tool, users can characterize candidate exchangers.

Another bioinformatics tool that users can use to learn about sodium calcium exchangers is available under the *Phylogeny* link. At this page, users can examine interactive phylogenentic trees of representative exchangers from each of the three exchanger subgroups. The user can select the subtype from a drop-down menu and click submit to view a tree for that specific exchanger subtype. The resulting tree exhibits markings for the Phylum and clades which are denoted by colored arcs around the radial tree, and each tip can be clicked to reveal more information and also links to external databases for each exchanger. This feature enables users to explore the evolutionary history of an exchanger subtype and identify closely related exchangers to exchangers of interest.

**Fig. 3:**
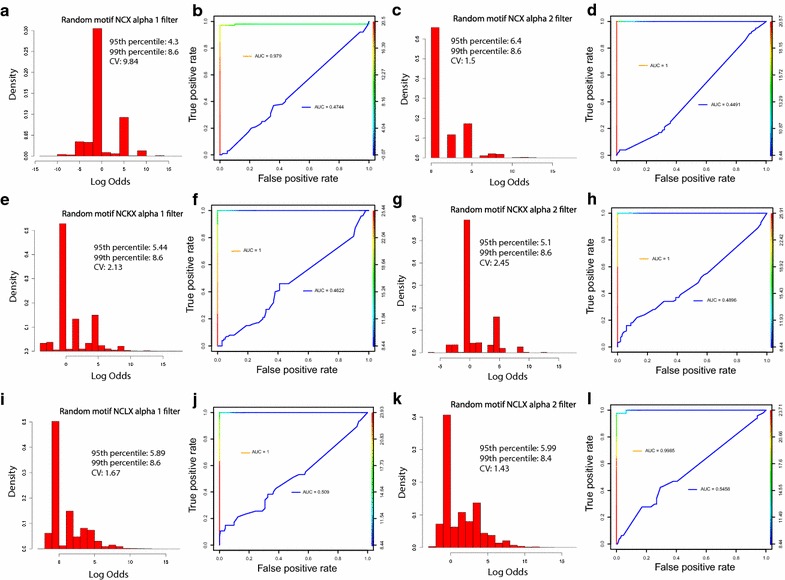
Comparison of NCX-DB exchanger classification to simulated random data. The classification feature of NCX-DB was benchmarked against a simulated dataset. In the NCX-DB filtering algorithm, NCX-DB searches for alpha repeat structures and filters candidates based upon log odds scores obtained for each motif. To benchmark this filtering algorithm, we compared the prediction accuracy of NCX-DB for a training dataset of known sodium calcium exchangers to a randomized data set. 100, 000 randomized alpha repeats were generated using a custom Perl script (Additional file 1). The density of log odds scores for each randomized alpha repeat is shown as histograms. **a** NCX alpha 1 repeat randomized data. 95th percentile = 4.3, 99th percentile = 8.6 coefficient of variation (CV) = 9.84; **c** NCX alpha 2 repeat randomized data. 95th percentile = 6.4, 99th percentile = 8.6, CV = 1.5; **e** NCKX alpha 1 repeat randomized data. 95th percentile = 5.44, 99th percentile = 8.6, CV = 2.13; **g** NCKX alpha repeat 2 randomized data. 95th percentile = 5.1, 99th percentile = 8.6, CV = 2.45; **i** NCLX alpha repeat 1 randomized data. 95th percentile = 5.89, 99th percentile = 8.6, CV = 1.67; **k** NCLX alpha repeat 2 randomized data. 95th percentile = 5.99, 99th percentile = 8.4, CV = 1.43. In all cases we observed the highest density of log odds scores close to zero. The 95th percentile log odds ranged from 4.3 to 6.4, and 99th percentile log odds ranged from 8.4 to 8.6. We then used this data to examine how well NCX-DB predicts the correct exchanger in a background of randomized data. The ROC curves in each case revealed AUC values close or at 1 for each motif: **b** NCX alpha 1 repeat AUC = 0.979 and random NCX alpha 1 repeat AUC = 0.4744; **d** the NCX alpha 2 repeat AUC = 1 and random NCX alpha 2 repeat AUC = 0.4491; **f** NCKX alpha 1 repeat AUC = 1 and random NCKX alpha 1 repeat AUC = 0.4622; **h** NCKX alpha 2 repeat AUC = 1 and random NCKX alpha 2 repeat AUC = 0.4896; **j** NCLX alpha 1 repeat AUC = 1 and random NCLX alpha 1 repeat AUC = 0.509; **l** NCLX alpha 2 repeat AUC = 0.9985 and random NCLX alpha 2 repeat AUC = 0.5458. Histograms were generated using the *hist(x,…)* function in R. ROC curves were made in R using the ROCR package (https://rocr.bioinf.mpi-sb.mpg.de/) using the *perf ← performance(pred,”tpr”,”fpr”)* to plot true positive rate against false positive rate. Control randomized data was added (*add *=* TRUE*) to the experimental *colorized* data

**Fig. 4:**
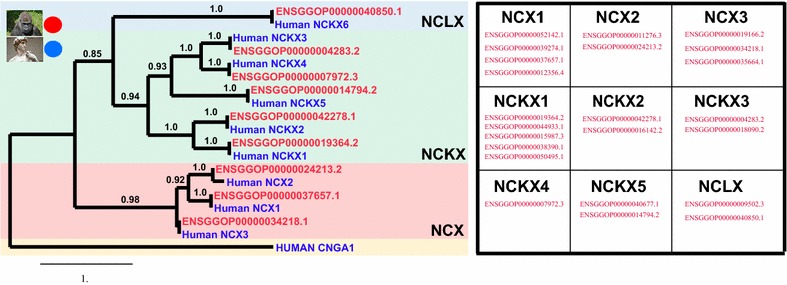
Identification and classification of sodium calcium exchangers from the *Gorilla gorilla* genome using NCX-DB. Phylogenetic tree constructed using PhyML [57] of the predicted canonical sodium calcium exchangers from *Gorilla gorilla* and human. The human cyclic nucleotide gated channel subunit, CNGA1, is used as an outgroup. 23 candidate sodium calcium exchangers isoforms were identified from Gorilla: 4 NCX1; 2 NCX2; 3 NCX3; 5 NCKX1; 2 NCKX2; 2 NCKX3, 1 NCKX4; 2 NCKX5; and 2 NCLX

A utility of NCX-DB and the *Predict* tool within NCX-DB is its ability to identify candidate exchangers and filter these exchangers into the appropriate exchanger subtype. In order to benchmark the ability of NCX-DB and *Predict* to identify and filter candidates appropriately, we compared our training data with a randomized data set which was generated using a customized Perl script (Additional file 1). In our filtering algorithm, NCX-DB searches for alpha repeat structures and filters candidates based upon log odds scores obtained for each motif. We generated a training data set comprised of known sodium calcium exchangers from mice, human, rat, *Drosophila melanogaster*, and *Caenorhabditis elegans*. The training data was only comprised of exchangers for which there is experimental and literature based evidence of functional NCX, NCKX or NCLX annotation. For our random data set we generated 100,000 random alpha repeat motifs for each exchanger type (Fig. 3) and plotted the density of their log odds scores (e.g. see Fig. 3a for random NCX alpha 1 repeat). In all cases we observed log odds scores around zero, which is what we would expect if the motifs were generated by chance. The 95th percentile log odds ranged from 4.3 to 6.4 (Fig. 3). Next, we compared the randomized data set with our training data to see how well NCX-DB predicts the correct exchanger in a background of randomized data. From this analysis, the ROC curves revealed AUC values close to 1 or at 1 for each motif: NCX alpha 1 repeat AUC = 0.979 and random NCX alpha 1 repeat AUC = 0.4744 (Fig. 3b); the NCX alpha 2 repeat AUC = 1 and random NCX alpha 2 repeat AUC = 0.4491 (Fig. 3d); NCKX alpha 1 repeat AUC = 1 and random NCKX alpha 1 repeat AUC = 0.4622 (Fig. 3f); NCKX alpha 2 repeat AUC = 1 and random NCKX alpha 2 repeat AUC = 0.4896 (Fig. 3h); NCLX alpha 1 repeat AUC = 1 and random NCLX alpha 1 repeat AUC = 0.509 (Fig. 3j); NCLX alpha 2 repeat AUC = 0.9985 and random NCLX alpha 2 repeat AUC = 0.5458 (Fig. 3l). These data revealed that NCX-DB predicts the correct exchanger in almost 100% of cases as compared with background simulated data. However, these tests do not take into account real background data, and nor do they consider how many exchangers NCX-DB may miss (i.e. false negatives). To address these concerns, we chose to use NCX-DB to predict exchanger subtypes within the Gorilla genome (*Gorilla gorilla*). We chose Gorilla for two reasons: firstly, a search in PubMed for “Gorilla” + “NCX” returns zero hits (as per 02/2018); therefore, little is known about the sodium calcium exchanger super-family in Gorilla and this makes it a good test case for determining how well NCX-DB can report exchanger subtypes from an unknown sample; secondly, we chose Gorilla because of its close phylogenetic relationship to humans. There has been a lot of work done on NCX from humans and therefore, we can hypothesis that the Gorilla genome should have at least the same number of exchanger subtypes as humans, and this will allow us to estimate the false negative rate for NCX-DB. We downloaded the genome of *Gorilla* (release 91) from *Ensembl* (https://useast.ensembl.org/Gorilla_gorilla/Info/Index), and used NCX-DB to identify candidate exchangers and filter them by exchanger subtype. We then aligned candidate exchangers using MUSCLE [56], and determined the best model of evolution from Prottest [60] before reconstructing the phylogenetic relationship of the candidate Gorilla exchangers with Human exchangers by maximum-likelihood using PhyML [57] (Fig. 4). In total, we identified 23 candidate exchangers from Gorilla. Four are predicted isoforms of NCX1, 2 are predicted isoforms of NCX2, and 3 are predicted isoforms for NCX-3. We identified 5 isoforms of NCKX1, 2 candidate isoforms of NCKX2, 2 candidate isoforms of NCKX3, a single NCKX4 ortholog, and 2 candidate isoforms of NCKX5. Finally, we also identified 2 candidate isoforms of NCLX (Fig. 4). The canonical isoforms for each subtype grouped perfectly with their human counterparts, with NCLX (NCKX6) forming a sister group to the NCKX subtypes (Fig. 4). These data provide clear evidence that the algorithm employed by NCX-DB can identify all the main exchanger subtypes from a genome with no published NCX exchanger experiments, and although there may still be exchangers that were missed, our algorithm clearly identified multiple isoforms for each exchanger subtype, with the exception of NCKX4 which was represented by a single protein. Overall, this suggests that our pipeline used to identify exchangers in NCX-DB returns almost no false positives, and based on the many isoforms predicted from the Gorilla genome, we conclude that at worst, NCX-DB exhibits a very low false negative rate. Although, more data would be needed to determine this rate more accurately.

Finally, when designing NCX-DB, we wanted to create a facility for researchers to stay up to date with new developments and literature as well as news in the field of sodium calcium exchangers. To accomplish this, we added a *Stats* page which provides a breakdown of data contained within NCX-DB and also a Twitter feed from a Twitter account which we set up for NCX-DB (Twitter handle @ncxdb). This Twitter account serves as an information center for everything related to sodium calcium exchangers and also a Twitterbot which automatically Tweets about new literature related to sodium calcium exchangers that come from PubMed (https://www.ncbi.nlm.nih.gov/pubmed), as well as the preprint servers: arXiv (https://arxiv.org/) and bioRxiv (https://www.biorxiv.org/). Twitter users can follow @ncxdb on Twitter or visit NCX-DB to stay up to date on new literature related to sodium calcium exchangers.

## Conclusions

NCX-DB is the first database of sodium calcium exchangers and provides a unified platform and suite of tools for researchers to compare exchangers across species while also discovering and characterizing new exchangers. To construct NCX-DB, large numbers of exchangers were sourced from several highly curated databases so as to provide the most up-to-date information of annotated exchangers in diverse species. We will test for updates each time one of the sourced databases issues a new release, and then provide updates to NCX-DB accordingly; these updates will be announced through Twitter and online. NCX-DB also contains a literature portal, where researchers can stay informed of recent publications and developments in the field of sodium calcium exchangers. In conclusion, NCX-DB will serve as a useful platform for exploration and discovery in future experiments on Na^+^/Ca^2+^ exchange biology.

## Availability of data

Project name: NCX-DB

Project home page: http://www.ncx-db.net

Operating system(s): Platform independent

Programming language: JavaScript, PHP, HTML, CSS

Other requirements: Modern Browser

License: GNU General Public License version 2, June 1991

Any restrictions to use by non-academics: None

## Additional file

**Additional file 1.** Random Peptide. A Perl script used to generate random peptides for histograms in Fig. 3.

## Abbreviations

NCX-DB: sodium calcium exchanger database
NCX: sodium calcium exchanger
NCKX: sodium calcium potassium exchanger
NCLX: sodium calcium lithium exchanger
CCX: calcium cation exchanger
JSON: JavaScript object notation
SQL: structured query language
PWM: position weight matrix
PPM: position probability matrix
NCBI: National Center for Biotechnology Information
BLAST: basic local alignment search tool
RSS: rich site summary
ROC: receiver operating characteristic
AUC: area under the curve
