# Supplementary material for: NCX-DB: a unified resource for integrative analysis of the sodium calcium exchanger super-family
Source: BMC Neurosci. 2018 Apr 13;19:19. doi: 10.1186/s12868-018-0423-2 (PMC5898058; doi:10.1186/s12868-018-0423-2)
Supplement: Supplementary file 1 — Additional file 1. Random Peptide. A Perl script used to generate random peptides for histograms in Fig. 3. [file 12868_2018_423_MOESM1_ESM.pdf]

```
#!/usr/bin/perl

use Modern::Perl;

#get peptide length and number of peptides from cmd-line
my $peptide_length = $ARGV[0];
my $num_peptides   = $ARGV[1];

#output file
my $outfileTxt = "random_peptides.txt";
open my $out, '>', $outfileTxt
  or die "Cannot open $outfileTxt: $!";

#array container for all peptides
my @random_proteins = makeRandomProteins( $peptide_length-1, $num_peptides-1 );

#loop through array and print each peptide on newline
while ( my $Element = shift(@random_proteins) ) {
    print $out $Element."\n";
}
#close FH;
close $out;

####SUBROUTINES
###############
sub makeRandomProteins {
    my $length = shift;
    my $number = shift;
    my $pep;
    my @result;

    foreach my $i ( 0 .. $number ) {
        $pep = RandomAA($length);
        push( @result, $pep );
    }
    return @result;
}

sub RandomAA {
    my $length = shift;
    my $pep;
    foreach my $i ( 0 .. $length ) {
        $pep .= randomAminoAcid();
    }
    return $pep;
}

sub randomAminoAcid {
```

```
    my (@amino_acids) = qw( A C D E F G H I K L M N P Q R S T V W Y );
    return randomResidue(@amino_acids);
}

sub randomResidue {
    my @array = @_;
    return $array[ rand @array ];
}
```
